# Role of Bioelectricity During Cell Proliferation in Different Cell Types

**DOI:** 10.3389/fbioe.2020.00603

**Published:** 2020-07-02

**Authors:** Mit Balvantray Bhavsar, Liudmila Leppik, Karla Mychellyne Costa Oliveira, John H. Barker

**Affiliations:** Frankfurt Initiative for Regenerative Medicine, Experimental Orthopedics and Trauma Surgery, J.W. Goethe University, Frankfurt, Germany

**Keywords:** membrane potential, V_mem_, cell proliferation, ouabain, mesenchymal stem cells, osteogenic sarcoma cells, fibroblasts

## Abstract

Most living organisms possess varying degrees of regenerative capabilities but how these regenerative processes are controlled is still poorly understood. Naturally occurring bioelectric voltages (like V_mem_) are thought to be playing instructive role in tissue regeneration, as well as embryonic development. The different distribution of ions on the either side of the cell membrane results in intra- and extra-cellular voltage differences, known as membrane potential or V_mem_. The relationship between V_mem_ and cell physiology is conserved in a wide range of cell types and suggests that V_mem_ regulation is a fundamental control mechanism for regeneration related processes e.g., proliferation and differentiation. In the present study we measured V_mem_ in three different cell types (human osteogenic sarcoma cell line (OSC), rat bone marrow derived mesenchymal stem cells (BM-MSC), and rat dermal fibroblasts) and characterized the relationship between their V_mem_ and proliferation. In order to find out if V_mem_ controls proliferation, or visa-versa, we blocked and then unblocked Na^+^/K^+^-exchanging ATPase using ouabain and measured the proliferation. Our results demonstrate that V_mem_ can be pharmacologically manipulated to control proliferation in certain cell types like BM-MSC. Taken together, it is clear that control of bioelectrical properties in non-excitable cells could prove to be potentially a useful tool in regenerative medicine efforts.

## Introduction

Current reconstructive treatments aimed at restoring normal form and function to diseased, injured or missing tissues and/or organs use a patient's own tissues, tissues and organs transplanted from donors, or prosthetic devices. While these treatments enjoy varying degrees of success, they are often associated with drawbacks such as limited donor availability, infection, immunological rejection, and high costs (Mao and Mooney, 2015). In contrast, regenerative therapies could potentially restore normal tissue form and function, without these drawbacks (Levin and Stevenson, 2012; Bessonov et al., 2015; Tyler, 2017). While most living organisms possess varying degrees of regenerative capabilities, the signals that control these processes are still poorly understood. Naturally occurring bioelectric signals have been shown to play an important role in tissue regeneration, as well as embryonic development (Gurtner and Chapman, 2016; Tyler, 2017).

**Figure 1:**
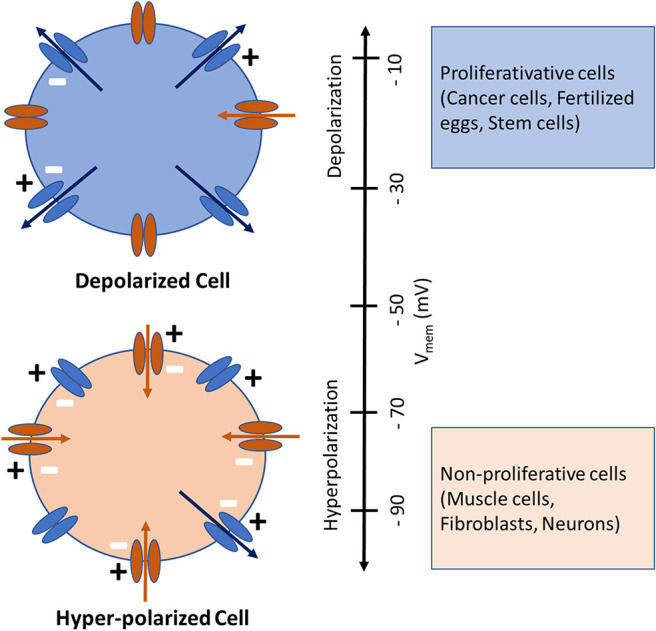
Depolarized (above) and Hyperpolarized (below) cells with ion channels shown schematically. The inward-rectifying channels (inward arrows) favor hyperpolarization while the outward-rectifying channels (outward arrows) favor depolarization. Cells physiological state is coinciding with its V_mem_, whereby highly proliferative (cancer, fertilized eggs, stem) cells are depolarized and mature, terminally differentiated (muscle, fibroblasts, neurons) cells are hyperpolarized. Image modified from Cervera et al. (2016) and Levin et al. (2019).

Bioelectricity originates at the cell membrane from a constant imbalance in charge between the intra- and extracellular compartments, caused by the passage of ions (Na^+^, K^+^, Ca^2+^, Cl^−^, etc.) through different types of ion pumps and channels. The different distribution of these ions on either side of the cell membrane results in intra- and extra-cellular voltage differences, known as membrane potential or V_mem_ (Levin et al., 2019). Such a balance is maintained via passive and active ion transport through various ion channels and transporters located within the membrane (Sundelacruz et al., 2009). Membrane potential forces ions to passively move in one direction: positive ions are attracted by the “negative” side of the membrane and negative ions by the “positive” one (Hammond, 2015). If we suppose that there is no concentration gradient for any ions (there is the same concentration of each ion in the extracellular and intracellular media), ions will diffuse according to membrane potential only positively charged ions, the cations Na^+^, Ca^2+^ and K^+^, will move from the extracellular medium to the intracellular one according to membrane potential. In contrast, anions (Cl^−^) will move from the intracellular medium to the extracellular one. V_mem_ is expressed relative to the extracellular environment so that a cell is “depolarized” when its V_mem_ is less negative, while a cell is “hyperpolarized” when its V_mem_ is more negative (Cervera et al., 2016; Erndt-Marino and Mariah, 2016). Accordingly, V_mem_ values of rapidly proliferating embryonic and tumor cells, generally have high “depolarized” V_mem_ values, whereas non-proliferating, terminally differentiated somatic cells, such as, skeletal muscle cells, neurons and fibroblasts typically have low “hyperpolarized” V_mem_ values as shown in Figure 1 (Binggeli and Weinstein, 1986; Chernet and Levin, 2013; Levin et al., 2019; Sundelacruz et al., 2019).

The relationship between V_mem_ and –cell physiology is conserved in a wide range of cell types (precursor and mature cells; proliferative and quiescent cells; normal and cancerous cells) and suggests that V_mem_ regulation is a fundamental control mechanism for regeneration related processes e.g., proliferation and differentiation (Sundelacruz et al., 2008, 2009). From this arises the intriguing possibility of being able to control a cell's proliferative and/or regenerative capabilities by manipulating its V_mem_. In other words, by increasing the V_mem_ of a normally non-proliferative fibroblasts, one could stimulate it to proliferate. Or conversely, by lowering the V_mem_ of a tumor cell one could reduce its proliferation and arrest its growth. In the present study we measured V_mem_ in three different cell types (osteogenic sarcoma cell line (OSC), bone marrow derived mesenchymal stem cells (BM-MSC), and fibroblasts) whose normal proliferative states are relatively high, medium and low, in order to characterize the relationship between their V_mem_ and proliferation. In order to investigate whether V_mem_ controls proliferation, or vise-versa, we blocked and then unblocked Na^+^/K^+^-exchanging ATPase and measured the proliferation.

## Materials and Methods

All experiments were performed in accordance with guidelines established by our animal care and oversight committed at the Johann Wolfgang Goethe University in Frankfurt am Main, according to German animal welfare act §4 and EU Act 2010/63 for the protection of laboratory animals.

### Cell Preparation and Culture

Rat BM-MSC were purchased from Cyagen Biosciences (Santa Clara, CA, USA Cat. No. RASMD-01001), fibroblasts were extracted from the skin of already euthanized rats (Seluanov et al., 2010) received from different project (German animal welfare act §4 and EU Act 2010/63), and human OSC cell line was purchased from the DMSZ-Cell bank (Braunschweig, Germany, Cell line: SAOS-2). All cells were stored in liquid nitrogen at −196°C, then, on the day of the experiment they were thawed, cultured, and expanded to reach the desired number. To achieve the appropriate number, cells were cultured until they reached 80% confluency and then expanded over 6–8 passages. Cells were then seeded in normal cell growth medium (Dulbecco's Modified Eagle Medium, GlutaMAX 1 g/L D-Glucose, 10% Fetal Calf Serum, and 1% Penicillin/Streptomycin (10 U/ml), all obtained from GibcoR (Gaithersburg, MD, USA), in 6-well cell culture plates (TPP, Trasadingen, Switzerland) at a density of 50,000 cell/cm^2^. All cells were cultured for 14 days in a humidified incubator at 37°C with 5% CO_2_, and culture medium was changed every 3 days.

### Na^+^/K^+^-Exchanging ATPase Blocker

Na^+^/K^+^-exchanging ATPase blocking was achieved using ouabain (10 μM, Sigma-Aldrich) which was added to the medium from a fresh stock solution in distilled water. This blocking effect was reversed (unblocked) by washing the cells 5 times with 1X PBS.

### Experimental Design

Each cell type was divided into two groups, (1) Cells with no blocker (control); (2) Cells with Na^+^/K^+^-exchanging ATPase blocker (ouabain). All cells/groups were cultured for 14 days during which time measurements were performed on days 0, 3, 5, 7, 10, and 14.

### V_mem_ Measurements

To visualize and measure V_mem_ changes at predetermined measurement time points (0, 3, 5, 7, 10, and 14 days) during proliferation, cells were dyed with the anionic voltage-sensitive dye, Bis-(1,3-diethylthiobarbituricacid) trimethine Oxonol [DiBAC_4_(3), Invitrogen, Carlsbad, CA, USA], whose uptake by cells is voltage dependent. Higher dye uptake is seen in more depolarized cells (Adams and Levin, 2014; Bhavsar et al., 2019). V_mem_ changes were visualized and measured using fluorescence microscopy, as described by Adams and Levin (2014). For each measurement a fresh solution of 10 mM DiBAC_4_(3), in DMSO was prepared and diluted to 0.5 mM in Hank's Buffered Salt Solution (HBSS, Invitrogen, Carlsbad, CA, USA). After adding the dye, the cells were left for 30 min in an incubator at 37°C, then washed two times using PBS at room temperature and imaged using a Nikon Eclipse Ti-E Inverted Microscope (Nikon, Tokyo, Japan). The DiBAC_4_(3) dye was excited with a 420 nm light and the fluorescence images were captured at 520 nm by a non-descanned photomultiplier tube, controlled by NIS Element Software. The captured images were saved as bright field (BF) images and for every BF image, a flatfield image (FF) (made by defocusing the image) and a dark field (DF) image (made by closing the shutter) were taken. These three images were later used for corrections and analysis (described in detail in Bhavsar et al., 2019). All samples were imaged on the same day to minimize time dependent variations. Since fluorescence intensity was quantified for each image, the gain, exposure time, and offset settings of the microscope were kept constant over the duration of each experiment.

### Cell Proliferation

To measure cell proliferation, cell number was evaluated using PicoGreen assay according to the manufacture's protocol (Quant-iTTM PicoGreen, ThermoFisher, Germany) at days 0, 3, 5, 7, 10, and 14. Briefly, cells were washed two times with PBS, treated with lysis buffer (400 mM potassium phosphate buffer, 2% Triton X100, 10 mM EDTA, pH 7.0), and cell lysates were used for DNA content measurements. A serial dilution of a known number of cells was lysed with lysis buffer and used to create a calibration curve showing the correlation between cell number and fluorescence. This latter procedure allowed us to indirectly determine the number of cells in the cultured wells via a calibration curve and measurement of DNA content through fluorescence of Pico-green.

### Cell Viability

To measure cell viability, AlamarBlue assay was performed at days 0, 3, 5, 7, 10, and 14. The AlamarBlue Assay incorporates an oxidation-reduction (REDOX) indicator that changes color in response to chemical reduction of growth medium resulting from cell growth. As cells being tested grow, innate metabolic activity results in a chemical reduction of AlamarBlue (resazurin) to resorufin. AlamarBlue assay was performed according to the manufacturer's protocol (AlamarBlue® Cell proliferation assay Kit, BIORAD). Briefly, the culture medium was completely aspirated from the wells and cells were washed twice with sterile PBS. One milliliter of fresh medium was added along with 100 μl of AlamarBlue reagent. Additionally, wells containing medium and AlamarBlue reagent only (no cells) were used for blank measurements. Cells and blank samples were incubated for 4 h (37°C, 5% CO_2_). After 4 h incubation, three aliquots (100 μl) of each sample were pipetted in a 96 well plate and absorbance was measured at 570 and 600 nm using plate reader (Infinite 200PRO Tecan, München, Germany). Absorbance mean values of triplicates for each sample were calculated and the percentage of Alamar blue reduction was calculated using a formula described in the manufacturer's protocol (AlamarBlue® Cell proliferation assay Kit, BIORAD).

### Data Analysis and Statistics

All experiments were performed in triplicate and the data is presented as the box and whisker plots unless otherwise indicated. The distribution of the data was checked using Shapiro-wilk test (*p* < 0.05 = non-parametric, *p* > 0.05 = parametric). The statistical significance of differences between the groups and time points was analyzed by non-parametric Friedman test and a Bonferroni corrected *p* < 0.05 was used to indicate statistical significance. The *p*-values are indicated on the plot using asterisks (^*^p < 0.05, ^**^p < 0.01). Statistics were calculated using the software Bias 11.03 (Epsilon-Verlag, Darmstadt, Germany).

## Results

### V_mem_ Profiles of Different Cell Types

**Figure 2:**
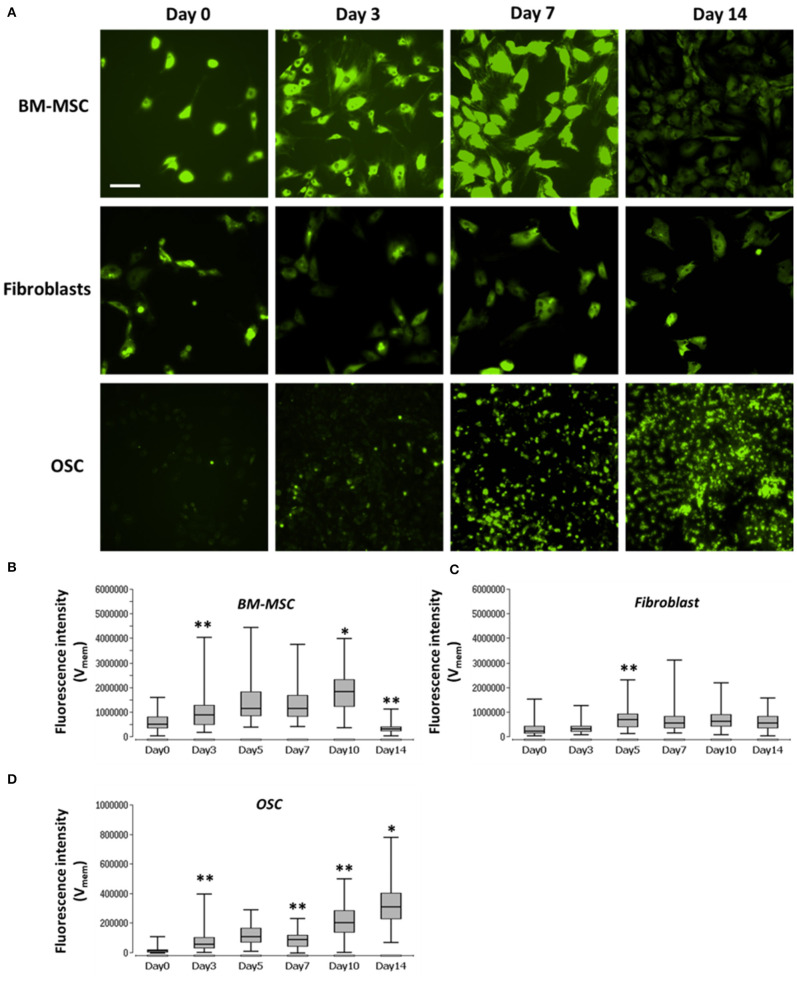
Images and graph of BM-MSC, fibroblasts, and OSC V_mem_ levels. **(A)** Representative fluorescence images of V_mem_ in BM-MSC, Fibroblasts and OSC at days 0, 3, 7, and 14. **(B)** V_mem_ (fluorescence intensity), of BM-MSC, **(C)** fibroblasts and **(D)** OSC at days 0, 3, 5, 7, 10, and 14 represented using Box and whisker plots (*n* = 114–135, 5–10 cells/Image from 15 images). Scale bar = 200 μm. Asterisks indicate degree of significant differences between groups at the same time points, *p < 0.05, **p < 0.01.

V_mem_ was measured in the three different cell types using voltage-sensitive dye DiBAC_4_(3) at different time points, as shown in Figure 2. V_mem_ values (fluorescence intensity) of BM-MSC increased from day 0 to day 10, significant at day 3 and 10 (*p* < 0.05). However, V_mem_ values significantly decreased (*p* < 0.01) at day 14. V_mem_ in fibroblasts, was constant during the entire time course. V_mem_ values in OSC increased, throughout, from day 0 to 14, significant at day 3, 7, 10, and 14 day 10 (*p* < 0.05) (see Supplementary Table 1 for details).

### Cell Proliferation and V_mem_ Measurements

**Figure 3:**
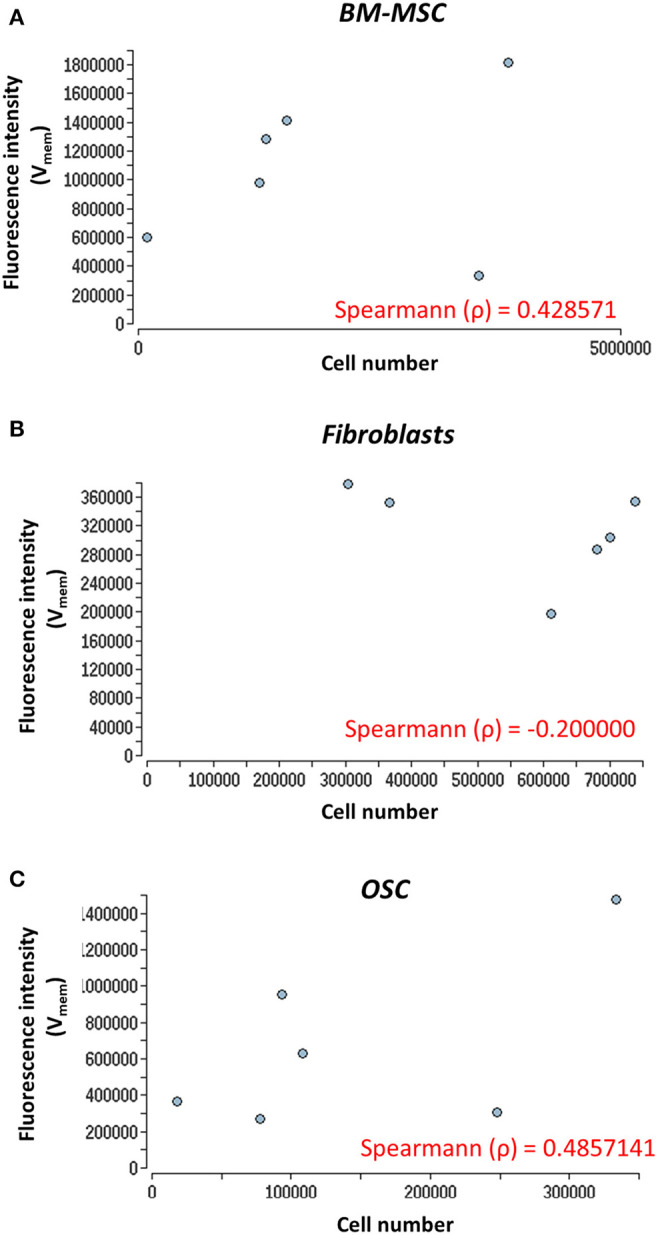
Nonparametric Spearman correlation analysis. Correlation between cell proliferation (cell number) and V_mem_ measurements. **(A)** BM-MSC, **(B)** fibroblasts, and **(C)** OSC proliferation (cell number) measured via fluorescence intensity (V_mem_) at days 0, 3, 5, 7, 10, and 14. The Spearman correlation coefficient (ρ) is indicated. The V_mem_ values are taken from Figures 2B–D.

Cell proliferation and V_mem_ in all three cell types were measured using PicoGreen assay and DiBAC_4_(3) voltage-sensitive fluorescent dye, respectively. Further, to determine the correlation, if any, between the cell proliferation and V_mem_, non-parametric Spearman correlation analysis was performed (Figures 3A–C). A moderate correlation between the cell number and V_mem_ was observed in BMMSC (ρ = 0.42) and in OSC (ρ = 0.48). However, no correlation (ρ = −0.2) was found between the cell number and V_mem_ in fibroblasts (Figure 3B) (see Supplementary Table 2 for details).

### Na^+^/K^+^-Exchanging ATPase Blocking and Cell Proliferation

**Figure 4:**
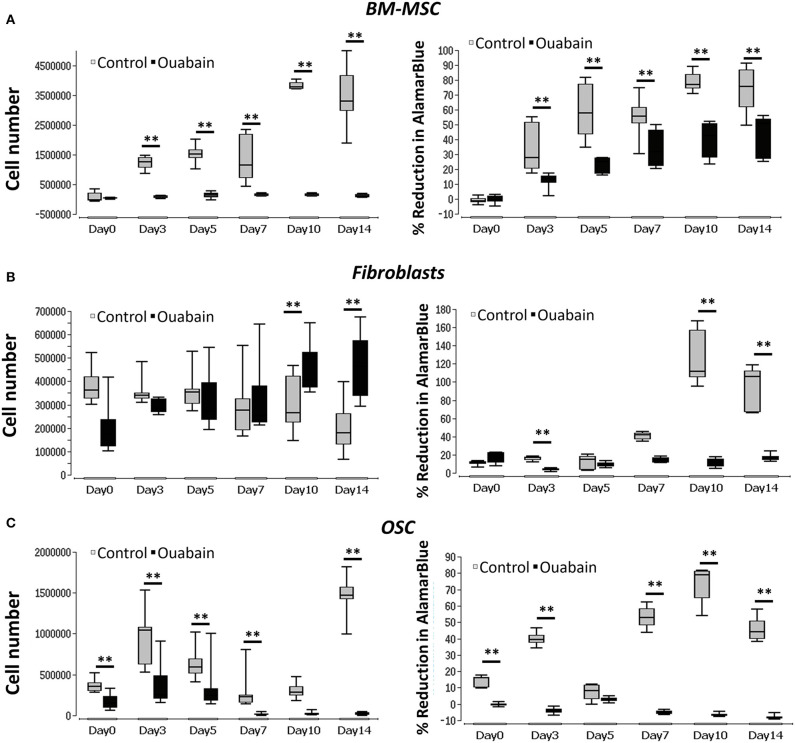
Graphs of BM-MSC, Fibroblasts, and OSC V_mem_ blocking (Na+-K+ ATPase) and cell proliferation. **(A)** Left- BM-MSC proliferation (cell number) measured in ouabain blocked and non-treated controls. Right- BM-MSC cell metabolic activity (% reduction of Alamar blue) measured in ouabain blocked and non-treated controls. **(B)** Left- fibroblasts proliferation (cell number) measured in ouabain blocked and non-treated controls. Right- fibroblast cell metabolic activity (% reduction of Alamar blue) measured in ouabain blocked and non-treated controls. **(C)** Left- OSC proliferation (cell number) measured in ouabain blocked and non-treated controls. Right- OSC cell metabolic activity (% reduction of Alamar blue) measured in ouabain blocked and non-treated controls. Asterisks indicate degree of significant differences between groups at the same time points. **p < 0.01. The cell numbers for control values are taken from Figure 3.

Na^+^/K^+^-exchanging ATPase was blocked, using ouabain, in all three cell types. During the blocking period, cell proliferation and viability were measured using PicoGreen and AlamarBlue, respectively. In the case of BM-MSC, the control group showed a significant increase (*p* < 0.01) in proliferation (cell number) from day 0 through 14, while cells treated with ouabain, showed neither increase nor decrease in the cell number (Figure 4A, left graph). In addition, BM-MSC V_mem_, blocked with ouabain, showed a significant reduction (*p* < 0.01) in cell metabolic activity at all the time points (Figure 4A, right graph). In contrasts, fibroblasts treated with ouabain, showed an increase in the cell number (significant at day 10 and day 14, *p* < 0.01) compared to their respective controls (Figure 4B). OSC treated with ouabain, showed a significant (*p* < 0.01) decrease in the cell number, especially at days 3, 5, 7, and 14 (Figure 4C, left graph). In addition, in ouabain treated OSC metabolic activity was significantly reduced (*p* < 0.01) at days 0, 3, 7, 10, and 14 (Figure 4C, right graph). The negligible negative values seen in OSC treated with Ouabain (Figure 4C, right) at later time points is due to the cytotoxic effect of Ouabain and drastic reduction in the cell number at later time points (Figure 4C, left graph). Apparently, there is almost no cells in these group at Day 10, 14 timepoints and therefore it is only % reduction of the culture medium. The calculations of % of reduction is always made against the negative control (medium only) samples and obtained negative values could be result of differences among mediums (pH) in samples and negative control wells since AlamarBlue is influenced by the pH of the cell growth medium (Rampersad, 2012) (see Supplementary Tables 3, 4 for details).

### Unblocking Na^+^/K^+^-Exchanging ATPase in BM-MSC

**Figure 5:**
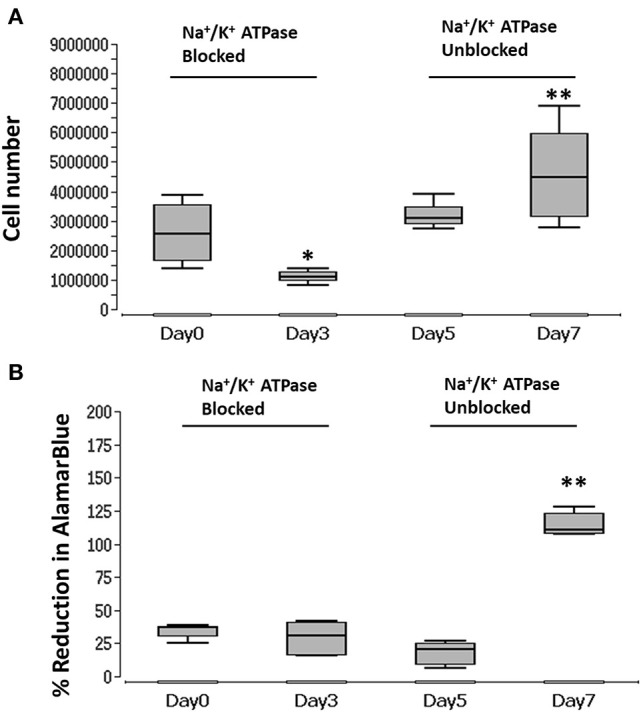
Graph of blocked and unblocked Na^+^/K^+^-exchanging ATPase of BM-MSC and proliferation. **(A)** BM-MSC proliferation (cell number) measured during blocking (ouabain) at days 3 and 5, and after unblocking (washing) at days 5 and 7. **(B)** BM-MSC cell metabolic activity (% reduction of Alamar blue) measured during blocking (ouabain) of Na^+^/K^+^-exchanging ATPase at days 3 and 5 and unblocking (washing) at days 5 and 7. Asterisks indicate degree of significant differences between groups at the same time points, *p < 0.05, **p < 0.01.

In order to unblock Na^+^/K^+^-exchanging ATPase, reversing the blocking effect of ouabain, BM-MSC were first treated with ouabain until day 3 and then washed five times using 1X PBS. The cell number and metabolic activity were visualized and measured at days 0, 3, 5, and 7 using PicoGreen and AlamarBlue, respectively. During the blocking phase, cells displayed significant (*p* < 0.05) reduction in number at day 3 with reduced (though not significant) cell metabolic activity (Figures 5A,B). After unblocking (washing with PBS), cell numbers were increased at days 5 and significantly at (*p* < 0.01) day 7. In addition, cell metabolic activity was significantly (*p* < 0.01) increased at day 7 (Figures 5A,B) (see Supplementary Table 5 for details).

## Discussion

The complex processes of tissue development, healing and regeneration involve multiple cellular activities like, proliferation, migration, adhesion and differentiation, all of which are, at least partially, regulated by V_mem_ related bioelectric signaling (Sundelacruz et al., 2008, 2009; Levin et al., 2019). In this study, we characterized membrane potential (V_mem_) profiles for BM-MSC, fibroblasts, and OSC during proliferation using the voltage-sensitive fluorescent dye DiBAC_4_(3). We observed that changes in V_mem_ and proliferation coincided, and that by blocking and unblocking Na^+^/K^+^-exchanging ATPase we were able to control proliferation in BM-MSC. We saw that as the cells began to proliferate, their V_mem_ values shifted from lower to higher (depolarization), and when they stopped proliferating, their V_mem_ shifted from higher to lower values (hyperpolarization). These observations coincided with those of others in the literature, which indicate that V_mem_ depolarization is required for both G1/S phase and G2/M phase transitions during cell proliferation (Sundelacruz et al., 2008; Blackiston et al., 2009; Yang and Brackenbury, 2013). In our experiments, OSC showed a consistent increase in V_mem_ during the course of proliferation. Reports in the literature suggest that cancer cells tend to be more depolarized than other cell types, due to a higher concentration of Na^+^/K^+^-exchanging ATPase (Yang and Brackenbury, 2013) and higher intracellular Na^+^ levels in comparison to other cell types (Camero et al., 1980; Sparks et al., 1983). This could explain the increase in V_mem_ we observed throughout the course of proliferation. On the other hand, we saw that fibroblasts V_mem_ displayed a pattern of hyperpolarized during the course of proliferation. This could be due to a lower number of voltage-gated Na+ channels present in dermal fibroblasts (Estacion, 1991), leading to lower concentrations of intracellular Na^+^, lower proliferative capacity and hyperpolarized V_mem_.

Pharmacological blocking of ion channels has been a popular method to study V_mem_ function. Using small molecule drugs to target specific ion channels of cells allows the precise control of a given cells' V_mem_ profile (Blackiston et al., 2009; Levin et al., 2019). Another advantage of pharmacological blocking over other methods (knockout, RNAi or morpholinos) is this approach can reveal membrane potential function *per se*, which is not necessarily dependent on any one particular gene (Blackiston et al., 2009). In this study we used ouabain, that affects V_mem_ by specifically blocking Na^+^/K^+^-exchanging ATPase or the sodium-potassium ion pumps in the cell membrane. Ouabain is a plant derived cardiac glycoside that was traditionally used in Africa as an arrow poison for hunting (Schoner, 2000). Ouabain blocks Na^+^/K^+^-exchanging ATPase by binding to the α-subunit at the external end of the ion permeation pathway and inhibits its ion exchange pump activity. Once ouabain binds to this enzyme, the pump ceases to function, leading to an reducing the activity of sodium-potassium ion pump which pumps one calcium ion out of the cell and three sodium ions into the cell (Shen et al., 2019).

In our experiments, we found that using ouabain to block Na^+^/K^+^-exchanging ATPase significantly decreased proliferation and reduced cell metabolic activity in BM-MSC. In order to confirm this finding, we subsequently unblocked the Na^+^/K^+^-exchanging ATPase, reversing Ouabain's effect and restoring proliferation and cell metabolic activity to their pre-blocked levels. This finding demonstrates that, by increasing or decreasing these cell's V_mem_, in this case using pharmacological blockers, one can control their proliferative capacity. In summary, our results suggest that pharmacological blocking/unblocking of Na^+^/K^+^ ATPase may provide a pro-proliferative environment in therapeutic tissue engineering applications where BM-MSC are used. Ouabain, that blocks Na^+^/K^+^ ATPase, is commonly used to treat congestive heart failure and supraventricular arrhythmias (Wu et al., 2015), however, its use systematically, in tissue engineering (TE) applications is unlikely. The growing field of TE, employs a large range of novel strategies, of which local ouabain application might be considered. For example, pre-treating cells *in-vivo* before transplanting them into a defect (Carpizo et al., 2008). Another approach could be to delivery ouabain into a defect incorporated in modified scaffolds, which release the drug locally and in a controlled manor (Kretlow et al., 2007; Garg et al., 2012; Sengupta and Prasad, 2018). Further animal studies are needed to determine whether ouabain's effects are maintained *in-vivo* in these conditions, and to demonstrate proof-of-concept using these approaches.

In dermal fibroblasts we found that using ouabain to block, initially (days 0–7) had no effect on proliferation, and then at days 10 and 14 blocking Na^+^/K^+^-exchanging ATPase in the cells caused an increase in proliferation, compared to controls. This finding coincides with those of others who reported that lower concentration of ouabain can induce proliferation in several different cell types, including fibroblasts (Orlov et al., 1999; Isaev et al., 2000; Nguyen et al., 2007; Winnicka et al., 2010). These authors propose that at lower concentrations ouabain can activate mitogen-activated protein kinase (MEK) and extracellular signal–regulated kinases (ERK) pathways, which in turn results in the expression of genes that are involved in cell growth and cell proliferation. Since ouabain can readily activate proliferation in dermal fibroblasts, it may have a direct impact on the dermal equivalents and bilayer skin substitutes which are used to treat a range of different chronic non-healing wounds in clinical settings (Wong et al., 2007).

We found that in OSC, blocking Na^+^/K^+^-exchanging ATPase with ouabain induced cell death at the later time points (days 7, 10, 14). In a similar study, Chou et al. (2018) investigated the effect of ouabain on apoptotic cell death of human osteosarcoma-derived U-2 OS cells. Based on their finding they suggest that blocking Na^+^/K^+^-exchanging ATPase with ouabain induced S-G2/M phase cell-cycle arrest in osteosarcoma cells, which in turn induced apoptotic cell death by activating the caspase-dependent and -independent pathways, accompanied also by mitochondrial dysfunction. Our findings support this and provide important insight into the cytotoxic effects of ouabain on OSC. It is noteworthy that many studies have shown the potential of ouabain as a therapeutic agent against various cancers (Winnicka et al., 2008; Liu et al., 2013; Pongrakhananon et al., 2013; Chen et al., 2014; Xiao et al., 2017; Shen et al., 2019). Our findings confirm this showing that blocking Na^+^/K^+^-exchanging ATPase with ouabain decreased osteosarcoma cell proliferation, suggesting it could potentially be used to treat osteosarcoma.

Our *in-vitro* results demonstrate that V_mem_ can be pharmacologically manipulated to control proliferation in certain cell types like BM-MSC. If reproduced in *in-vivo* models this may be used to regulate specific cell behaviors in cell-based clinical therapies to optimize their effectiveness.

## Data Availability Statement

All datasets generated for this study are included in the article/Supplementary Material.

## Author Contributions

MB conceived and designed the experiments, performed the experiments, analyzed the data, prepared figures and/or tables, and assisted in preparing the manuscript for publication. LL conceived and designed the experiments, contributed reagents/materials/analysis tools, and assisted in preparing the manuscript for publication. KC assisted in preparing the manuscript for publication. JB edited and corrected the manuscript. All authors contributed to the article and approved the submitted version.

## Conflict of Interest

The authors declare that the research was conducted in the absence of any commercial or financial relationships that could be construed as a potential conflict of interest.

## Supplementary Material

Supplementary Table 1 Raw data of Vmem measurements in BMMSC, Fibroblasts, and OSC (Figure 2).

Supplementary Table 2 Raw data of Vmem and cell number for calculating Spearman correlation coefficient (Figure 3).

Supplementary Table 3 Raw data of AlamarBlue measurement in BMMSC, Fibroblasts, and OSC (Figure 4).

Supplementary Table 4 Raw data of pico-green measurement in BMMSC, Fibroblasts, and OSC (Figure 5).

Supplementary Table 5 Raw data of picogreen and AlamarBlue measurement for blocking-unblocking experiments in BMMSC (Figure 5).
